# Unilateral Open-Angle Glaucoma Associated with the Ipsilateral Nevus of Ota

**DOI:** 10.1155/2013/924937

**Published:** 2013-04-09

**Authors:** Lidija Magarasevic, Zihret Abazi

**Affiliations:** ^1^Glaucoma Services, Eye Clinic, Zvezdara University Medical Center, 161 Dimitrija Tucovica Street, 11000 Belgrade, Serbia; ^2^Department of Ophthalmology, Health Center “Palilula”, 16 Knez Danilova Street, 11000 Belgrade, Serbia

## Abstract

The nevus of Ota also known as “congenital melanosis bulbi” and “oculodermal melanocytosis” is a blue-gray hyperpigmentation that occurs on the face and eyes. The sclera is involved in two-thirds of cases (causing an increased risk of glaucoma). Women are nearly five times more likely to be affected than men. It is rare among Caucasian people. The nevus of Ota is congenital or acquired. Most cases of the nevus of Ota are unilateral (90%), although pigmentation is present bilaterally in 5%–10%. Ocular abnormalities included pigmentation of the sclera, cornea, retina, and optic disc and cavernous hemangiomas of the optic disc, elevated intraocular pressure, glaucoma, and ocular melanoma. We reported an appearance of unilateral glaucoma in a Caucasian female patient with the acquired, ipsilateral nevus of Ota.

## 1. Introduction

The nevus of Ota, also known by its original name “nevus fuscoceruleus ophthalmomaxillaris,” is a hamartoma of dermal melanocytes. Ota, known by the pen name Mokutaro Kinoshita, was a Japanese doctor who first described the condition in 1939 [1]. The nevus of Ota is usually located in the region of ophthalmic and maxillary division of the trigeminal nerve. The conjunctiva, sclera, cornea, retina, and optic disc may also be involved. The nevus of Ota may be associated with oral, uveal, or leptomeningeal melanosis, and malignant transformation has been reported repeatedly [2, 3]. The nevus of Ota is most frequent in Asian populations, with an estimated prevalence of 0.014%–0.034%; it is uncommon in Caucasian patients [4]. Glaucoma has also been associated with the nevus of Ota in 10% of patients [5]. In our work, we report an acquired form of Ota's nevus associated with open-angle glaucoma which was diagnosed five years after the appearance of typical blue-gray hyperpigmentation.

## 2. Case Report

**Figure 1:**
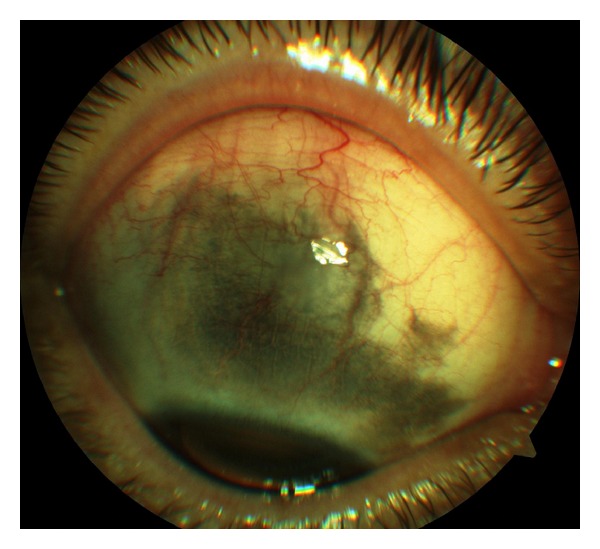
Slit-lamp photograph shows bluish-grey pigmentation of the right conjunctiva, episclera, and sclera.

A 47-year-old Caucasian female presented with a 5-year-history bluish-grey pigmentation of the right conjunctiva, sclera, and eyelids (Figure 1). As observed from her previous annual ophthalmic examination, the intraocular pressure never exceeded 16 mmHg, and the optic nerve head appeared normal in both eyes. Gonioscopy showed wide open and medium pigmented chamber angle with all elements of angle visible. Humphrey visual fields and optical coherence tomography findings did not show glaucoma damage. Her medical history included high blood pressure.

**Figure 2:**
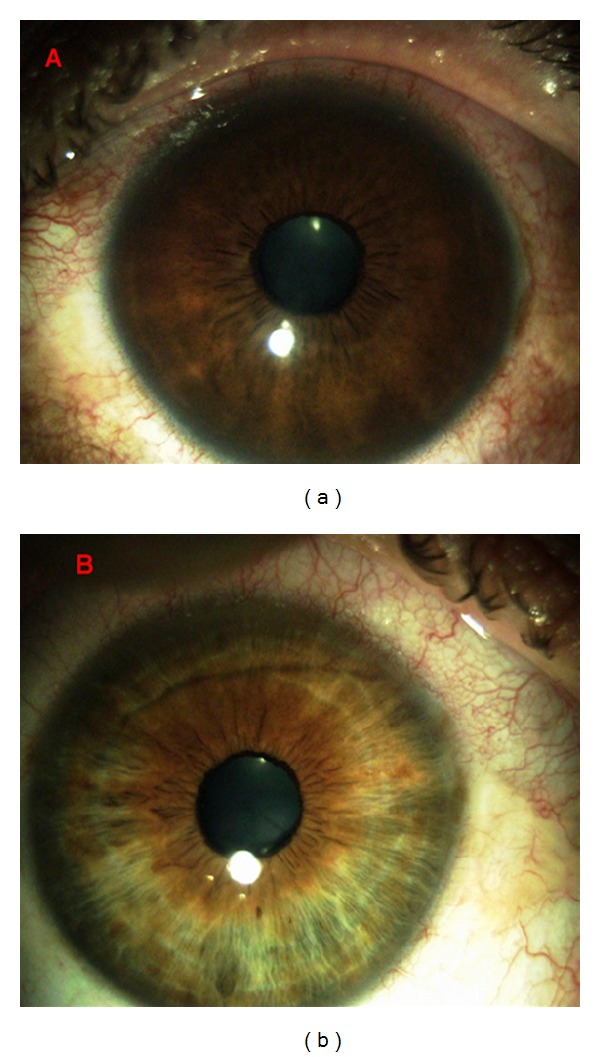
Slit-lamp photographs show folding on the iris, more prominent in the pupil area in the right eye (mammillations) (a). The darker iris in the right eye compared to the blue iris of the left eye (b) (heterochromia iridis).

**Figure 3:**
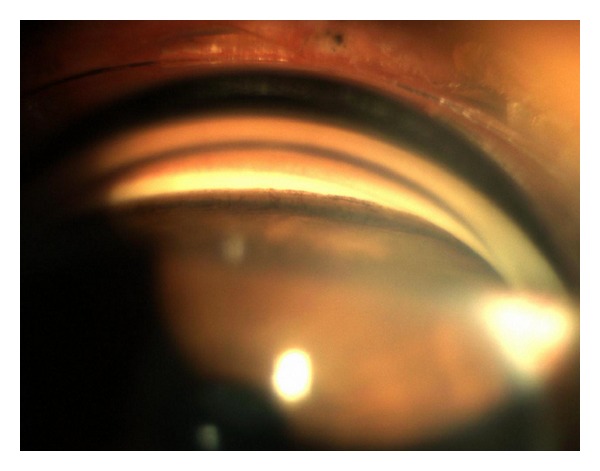
Gonioscopy of the right eye shows wide open and much pigmented chamber angle. The intense pigmentation on the external wall of the angle prevented the observation of other structures.

**Figure 4:**
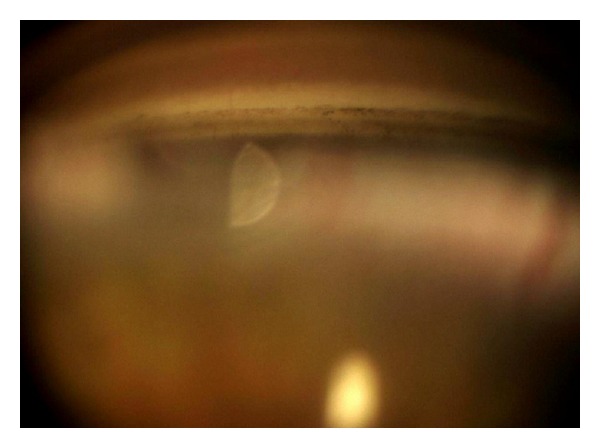
Gonioscopy of the left eye shows wide open chamber angle and with mild pigmentation.

**Figure 5:**
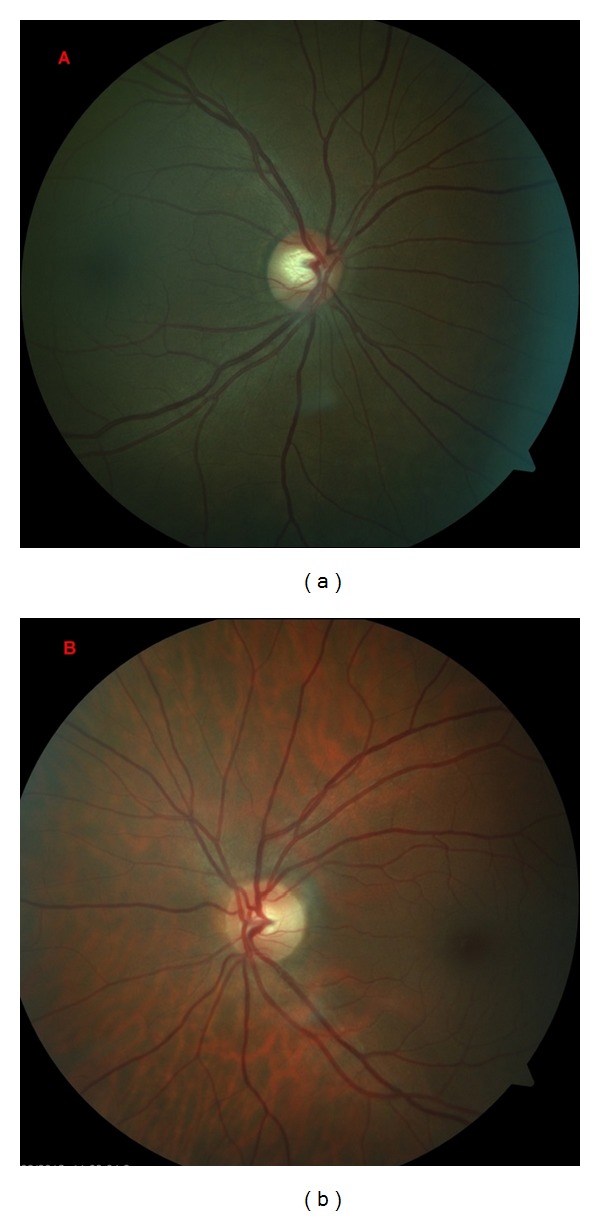
Fundus photography of the right eye (a) shows “darker fundus” (more pigment in the choroid) by ophthalmoscopy. The physical appearance of the right optic disc shows signs of glaucoma (C/D 0.4). Fundus photographs of the left eye (b), and optic head nerve is normal (C/D 0.2).

**Figure 6:**
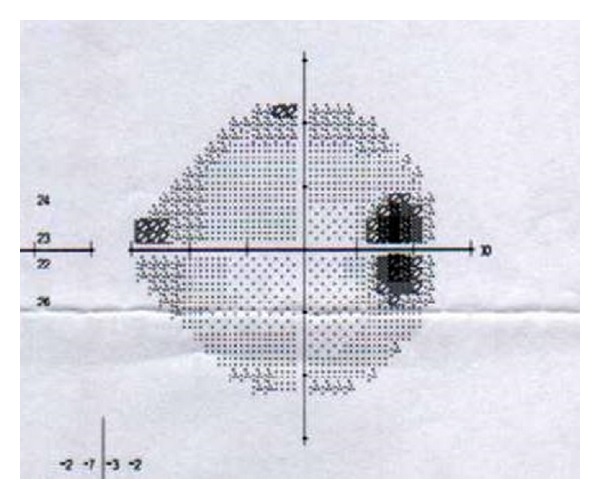
Humphrey visual field using the Swedish interactive thresholding algorithm Standard 24-2 program reveals defects that are typical for glaucoma. The overall sensitivity was reduced in right eye with a mean deviation of 3.97 dB.

The patient came for a regular annual ophthalmic exam. The visual acuity was 20/20 in both eyes. The IOP was measured in both eyes using a Goldmann applanation tonometer; diurnal IOP variations were 21–29 mmHg in the right eye and 12–16 mmHg in the left eye. In comparison to blue iris in the left eye, the right eye showed darker iris (heterochromia iridis) and numerous tiny pigmented nodules and folding on the iris (mammillations) (Figures 2(a) and 2(b)). Gonioscopy showed wide open and much pigmented chamber angle in the right eye than five years ago (Figure 3). In the left eye, chamber angle was wide open and not pigmented (Figure 4). In the right eye, the intense pigmentation on the external wall of the angle prevented the observation of other structures. Dilated fundus ophthalmoscopy demonstrated asymmetric “darker” pigmentation of the choroid layer beneath in the right eye (Figure 5(a)), which was not present in the left eye fundus (Figure 5(b)). Cup-to-disc ratio (C/D) in the right eye was 0.4, demonstrating glaucomatous damage to the optic head nerve; C/D was 0.2 in the left eye; and the optic head nerve was normal. In comparison to the normal visual field of the left eye, the Humphrey visual field of the right eye reveals an enlarged blind spot (Figure 6). SD OCT of the right eye reveals glaucomatous excavation of the head of the optic nerve with the thinning of RNFL in the superior and inferior parts. Eyelid biopsy revealed the pigmentation of the basal layer with elongated spindle shaped melanocytes in the dermis. Careful examination showed no systematic changes associated with the nevus of Ota. We also excluded neurological or any other cause of the optic head changes.

## 3. Discussion

The nevus of Ota primarily affects Asian populations. Other ethnic groups with increased prevalence include Africans, Afro-Americans, and East Indians. It is very uncommon among Caucasian patients. It has been rarely studied, although some studies report an estimated rate of about 0.038% of all the dermatological outpatient cases [6]. The nevus of Ota in this case was classified into a type IA (mild orbital type) by Tanino [7]. The occurrence of glaucoma in patients with the nevus of Ota may be related to the obstruction of aqueous outflow by accumulated melanocytes, but some authors suggest that their studies have not clearly shown that the Ota's nevus alone can cause the secondary glaucoma [8]. In its report, Cronemberger et al. indicate that there was no glaucoma in the eight eyes in which the gonioscopy revealed an external wall of the intensively pigmented angle [9]. Intensive pigmentation of the anterior chamber angle leads to the increase in IOP and glaucoma five years after the appearance of the nevus of Ota. Before glaucoma developed, anterior chamber angle was moderately pigmented. When the pigmentation of the chamber angle is intensive there is an increased risk of glaucoma associated with Ota's nevus, and the patient should be referred for periodic measurements of intraocular pressure and a complete ophthalmic examination.
